# Vitamin D Genomics: From *In Vitro* to *In Vivo*

**DOI:** 10.3389/fendo.2018.00250

**Published:** 2018-05-23

**Authors:** Carsten Carlberg

**Affiliations:** School of Medicine, Institute of Biomedicine, University of Eastern Finland, Kuopio, Finland

**Keywords:** vitamin D, vitamin D receptor, vitamin D target genes, vitamin D intervention trial, chromatin, epigenome, immune system

## Abstract

The vitamin D_3_ metabolite 1α,25-dihydroxyvitamin D_3_ [1,25(OH)_2_D_3_] is the exclusive high-affinity ligand of the vitamin D receptor (VDR), a transcription factor with direct effects on gene expression. Transcriptome- and epigenome-wide data obtained in THP-1 human monocytes are the basis of the chromatin model of vitamin D signaling. The model describes, how VDR’s spatio-temporal binding profile provides key insight into the pleiotropic action of vitamin D. The transcription of some 300 primary target genes is significantly modulated through the action of genomic VDR binding sites in concert with the pioneer transcription factor PU.1 and the chromatin organizer CTCF. In parallel, the short-term vitamin D intervention study VitDbol (NCT02063334) was designed, in order to extrapolate insight into vitamin D signaling from *in vitro* to *in vivo*. Before and 24 h after a vitamin D_3_ bolus chromatin and RNA were prepared from peripheral blood mononuclear cells for epigenome- and transcriptome-wide analysis. The study subjects showed a personalized response to vitamin D and could be distinguished into high, mid, and low responders. Comparable principles of vitamin D signaling were identified *in vivo* and *in vitro* concerning target gene responses as well as changes in chromatin accessibility. In conclusion, short-term vitamin D supplementation studies represent a new type of safe *in vivo* investigations demonstrating that vitamin D and its metabolites have direct effects on the human epigenome and modulate the response of the transcriptome in a personalized fashion.

## Introduction

The energy of sunlight-derived UV-B (290–315 nm) is used in human skin to convert the ubiquitous cholesterol precursor 7-dehydrocholesterol into pre-vitamin D_3_ that isomerizes in a non-enzymatic reaction to the secosteroid vitamin D_3_ (1). The hydroxylation of vitamin D_3_ to 25-hydroxyvitamin D_3_ [25(OH)D_3_] and then to 1,25(OH)_2_D_3_ is necessary to generate the biologically most active metabolite (2). Lifestyle choices, such as preferential indoor activities and coverage by textile outdoors, in combination with climatic and seasonal changes are the main reasons for insufficient UV-B exposure of the majority of today’s human populations (3). In order to avoid deficiency due to this low endogenous production, vitamin D_3_ needs to be taken up by diet or supplementation with pills.

Well-known physiological roles of vitamin D are (i) control of intestinal absorption of calcium and phosphorus from diet, (ii) renal reabsorption of calcium, and (iii) remodeling of bones (4). However, vitamin D and its receptor, the transcription factor vitamin D receptor (VDR), are involved in far more functions than maintaining calcium homeostasis and bone integrity (5). Highest *VDR* gene expression is found in metabolic tissues, such as intestine, kidneys, and bone, but low to moderate *VDR* levels can be observed in more than half of the some 400 tissues and cell types forming the human body (www.proteinatlas.org/ENSG00000111424-VDR/tissue). For example, vitamin D modulates the responsive of both the innate and the adaptive immune system, i.e., it supports the human body in its fight against infections and in parallel prevents autoimmune disorders (6). Accordingly, vitamin D deficiency results not only in problems with bones, which are rickets in children or increased fracture risk for adults, but also weakens vitamin D’s protective role of against diseases like tuberculosis, multiple sclerosis, and type 1 diabetes (7).

The lipophilic structure of vitamin D_3_ and its metabolites allows the molecules passing through biological membranes. Thus, gene regulation by vitamin D is more direct and less complex than that of peptide hormones, growth factors, cytokines, and other hydrophilic signaling molecules. Since VDR is the only protein binding 1,25(OH)_2_D_3_ with high-affinity (8), the physiological effects of vitamin D are largely identical to those of its receptor. Thus, comprehensive insight into vitamin D signaling requires the understanding of VDR’s molecular actions.

**Figure 1:**
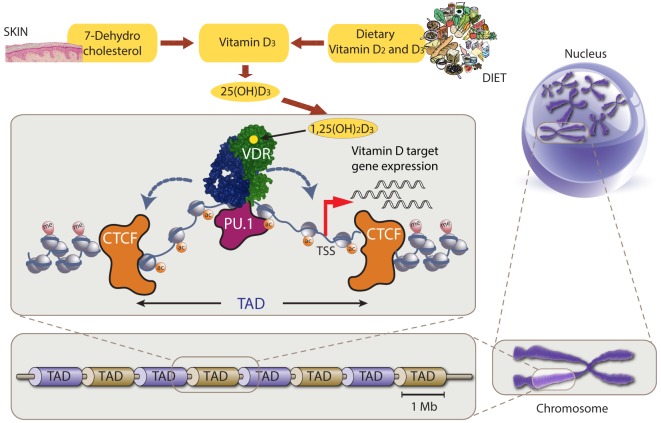
Chromatin model of vitamin D signaling. Top: production of vitamin D_3_ and its metabolites 25(OH)D_3_ and 1,25(OH)_2_D_3_. Center: vitamin D receptor (VDR) (green) binds accessible genomic DNA in complex with a partner protein (RXR or others, blue). VDR’s DNA binding is supported by the pioneer factor PU.1 (purple). The genomic region that can be influenced by 1,25(OH)_2_D_3_ (*via* binding to VDR) is restricted by CTCF proteins (orange) defining left and right topologically associated domain (TAD) borders, i.e., only vitamin D target genes within the TAD will be stimulated to produce more mRNA copies. Bottom and right: schematic illustration of TAD size on relation to chromosomes and the nucleus.

Vitamin D receptor belongs the nuclear receptor superfamily, most members of which are activated by small lipophilic molecules (9). Within VDR’s ligand-binding domain some 40 amino acids, which are mostly non-polar, form a ligand-binding pocket that fixes 1,25(OH)_2_D_3_ with high specificity and affinity (10). The binding of ligand induces a change in the conformation of the ligand-binding domain, so that VDR’s protein–protein interaction profile alters from that of a repressor to that of an activator (11, 12). Thus, VDR functions as a vitamin D-sensitive switch that attracts a set of nuclear proteins, like co-factors and chromatin modifying enzymes, to its thousands genomic binding sites (Figure 1). This leads to local changes in chromatin accessibility at many genomic loci, i.e., the epigenome responds to vitamin D.

The expression of a primary vitamin D target gene is modulated, i.e., in most cases increased, when it co-locates with a prominent VDR binding site within the same higher order chromatin structure, referred to as topologically associated domain (TAD) (13). An additional condition is that the transcription start site (TSS) of the vitamin D target gene and a VDR-binding enhancer region are within accessible chromatin (12). Thus, changes in the epigenome are the first events after stimulation of a cell with vitamin D before the transcriptome gets modulated.

This review describes a transition in the understanding of vitamin D signaling. The latter was on *in vitro* cell culture models and now gets new insights from *in vivo* investigations in the context of short-term vitamin D intervention trials.

## Genome-Wide VDR Binding Patterns *In Vitro*

During the past years, the method chromatin immunoprecipitation sequencing (ChIP-seq) was widely used for the description of the genome-wide VDR binding pattern, the so-called “VDR cistrome” (14). VDR ChIP-seq data have been obtained in a number of human *in vitro* cell culture models, such as GM10855 and GM10861 human B lymphocytes (15), LS180 colorectal cancer cells (16), LX2 hepatic stellate cells (17), and lipopolysaccharide-polarized THP-1 macrophage-like cells (18). In parallel, in mouse cells VDR ChIP-seq had been performed with 3T3-L1 pre-adipocytes (19), IDG-SW3 osteocytic cells (20), pre-osteoblastic and differentiated MC3T3-E1 osteoblastic cells (21), as well as with bone marrow-derived mesenchymal stem cells differentiating into bone and fat cells (22). However, the presently most comprehensive analysis of the spatio-temporal VDR binding pattern has been performed in undifferentiated THP-1 human monocyte-like cells (13, 23).

Cell culture models have the advantage of rather homogenous cell populations that mostly display an unlimited growth potential. This allows performing biological repeats without the risk of major variations. Moreover, growth media can be depleted from lipophilic molecules, such as vitamin D_3_ and its metabolites, so that a stimulation with pharmacologic doses of 1,25(OH)_2_D_3_ (10–100 nM) results in maximal induction in reference to untreated cells. Accordingly, VDR ChIP-seq datasets obtained from *in vitro* cell models unanimously demonstrate that stimulation with 1,25(OH)_2_D_3_ significantly increases the number of genomic VDR binding events 2- to 10-fold (18).

The cistrome of ligand-stimulated VDR comprises some 2,000–10,000 sites per cell type. The VDR binding pattern is rather cell-specific and only the small subset of some 50 sites is found in all investigated cell types (18). Therefore, most *VDR* expressing tissues and cell types have a rather different set of vitamin D target genes (14, 24).

In agreement with findings of the ENCODE project (25) VDR binds equally likely both up- and downstream of genes, i.e., VDR binding sites are distributed in a Gaussian fashion in relation to the TSSs of primary vitamin D target genes. Accordingly, the more distant VDR binding sites are from a TSS, the less likely they are functional for the respective gene. In addition, the VDR binding site within an enhancer region and the TSS of a primary vitamin D target gene under the control of the receptor have to be located within the same TAD. Interestingly, out of 11,600 VDR binding sites identified in THP-1 cells, the small subgroup of only 339 highly conserved persistent VDR loci is well suited for describing most vitamin D gene regulatory scenarios (13). In THP-1 cells almost all primary vitamin D target genes are located within 1,25(OH)_2_D_3_-modulated TADs (more details below). Conserved persistent VDR sites control 168 of the 311 primary vitamin D target genes, whereas 120 genes are close to transiently occupied VDR sites. The equal distribution of persistent VDR binding sites over the human genome suggests that they may be strategically positioned, in order to provide the whole genome with sensitivity to vitamin D (13). The similarly equal genomic distribution of primary vitamin D target genes (26) supports this concept. Thus, the time-dependent binding profile of a few 100 VDR loci is sufficient for regulating most primary vitamin D target genes.

## Chromatin Responses to 1,25(OH)_2_D_3_

Genomic DNA is not “naked” but always wrapped around nucleosomes forming a protein–DNA complex that is referred to as chromatin (Figure 1). Nucleosomes are composed of eight histone proteins that are rich in the basic amino acids lysine and arginine. In particular at the histone’s protruding amino-termini these amino acids are often post-translationally modified by methyl or acetyl groups. Such histone modifications alter the structure of chromatin by affecting the non-covalent interactions within and between nucleosomes.

**Figure 2:**
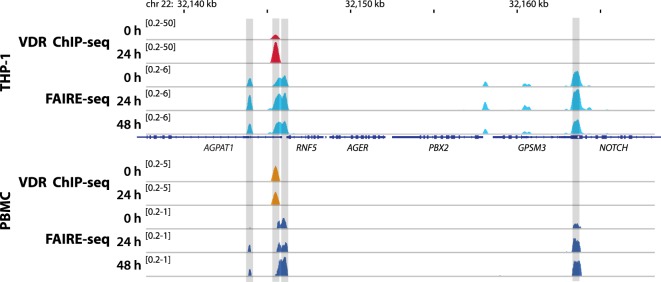
Vitamin D receptor (VDR) binding and chromatin opening of the 1-acylglycerol-3-phosphate O-acyltransferase 1 (*AGPAT1*) locus *in vitro* and *in vivo*. Top: THP-1 cells were stimulated for 0, 24, and 48 h with 1,25(OH)_2_D_3_ and VDR chromatin immunoprecipitation sequencing (ChIP-seq) and formaldehyde-assisted isolation of regulatory elements sequencing (FAIRE-seq) were performed. Bottom: in an analogous *in vivo* experiment (phase II context of the VitDbol study) one individual was challenged with a vitamin D_3_ bolus (2,000 µg). The average raise in 25(OH)D_3_ serum concentrations at days 1 and 2 after the vitamin D_3_ bolus was 11.9 and 19.4 nM, respectively. Peripheral blood mononuclear cells (PBMCs) were isolated before (day 0) and at days 1 (24 h) and 2 (48 h) and VDR ChIP-seq and FAIRE-seq were performed. The integrative genomics viewer browser was used to visualize the *AGPAT1* gene locus. The peak tracks represent mergers of each three biological repeats. Gene structures are shown in blue.

The epigenome is the genome-wide representation of (i) some 100 different histone marks, (ii) the level of DNA methylation at CpG islands, and (iii) higher order chromatin organization (27). The epigenome dynamically responds to extra- and intracellular signals, such as ligand activation of the VDR (28). However, most chromatin regions are intrinsically repressed, so that the binding of transcription factors and other nuclear proteins to genomic DNA is prevented, i.e., the epigenome controls the access to the genome (29, 30). In consequence, in a differentiated cell the so-called “epigenetic landscape” is restricted to some 100–200,000 chromatin loci (Figure 2 shows an example genomic region) that are accessible to transcription factors and RNA polymerases (25). This represents less than 10% of the whole chromatin and primarily refers to regions carrying TSSs and enhancers.

Chromatin modifying and remodeling enzymes read, write, or erase chromatin marks and reposition nucleosomes, respectively (31). These enzymes are modulated in their activity by signal transduction cascades originating from intra- and extracellular signaling molecules and/or form complexes with transcription factors specifically binding to respective genomic regions (32). VDR communicates with chromatin modifying enzymes *via* direct and indirect interaction, such as up- and down-regulating their genes (33) or being part of the same large protein complex in the nucleus (34).

On the genome-wide level, vitamin D-induced alterations in the chromatin accessibility profile can be measured *via* the method formaldehyde-assisted isolation of regulatory elements sequencing (FAIRE-seq, Figure 2). In THP-1 cells, FAIRE-seq identified 62,000 accessible chromatin loci, nearly 9,000 of which are significantly modulated by 1,25(OH)_2_D_3_ (28). A 2 h stimulation with 1,25(OH)_2_D_3_ resulted at more than 3,300 genomic loci in significant changes in chromatin accessibility, after 24 h even more than 4,500 sites responded, while after 48 h only some 2,400 regions were targets of vitamin D. This suggests that maximal epigenome-wide effects occur after 24 h. In parallel, this indicates that the process of chromatin opening by vitamin D includes multiple steps. Although the exact molecular mechanisms of these vitamin D-triggered epigenome changes are not fully understood, it is obvious that they are secondary consequences of genome-wide VDR binding.

## The Chromatin Model of Vitamin D Signaling

The human genome is subdivided into at least 2,000 chromatin loops (35), which segregate each chromosome into TADs. The latter are functionally independent chromatin subdomains in the size of hundreds of kilobases to a few megabases. Insulator regions separate TADs from each other (36) and contain binding sites for the transcription factor CTCF. This makes CTCF a key protein in organizing chromatin into active and inactive regions. However, from the 20,000 genome-wide CTCF loci, only some 15% are involved in forming TAD anchor regions. Interestingly, in THP-1 cells the binding of CTCF to some 1,300 sites is affected significantly by a stimulation with 1,25(OH)_2_D_3_ (37). More than half of the vitamin D-modulated CTCF sites mark one or both anchors of some 600 TADs, each of which comprises at least one VDR binding site and one vitamin D target gene. Interestingly, in the same cellular system, 587 genes are regulated significantly by 1,25(OH)_2_D_3_ (13).

In addition to the chromatin organizer CTCF, VDR also functionally associates with pioneer factors, such as PU.1 (38) or GABPA (39). A pioneer factor is a transcription factor that (i) displays many genomic binding sites, (ii) shows some promiscuity in DNA binding, and (iii) has a high diversity in protein–protein interactions (40). Accordingly, after a cellular perturbation pioneer factors are the first protein binding enhancers interacting with chromatin modifying enzymes. This makes chromatin more accessible for regular transcription factors like VDR. Interestingly, in THP-1 cells a 24 h stimulation with 1,25(OH)_2_D_3_ significantly modulated PU.1 binding at more than 5,600 sites (38).

In summary, in the THP-1 model system the epigenome-wide outcomes of a 24 h stimulation with 1,25(OH)_2_D_3_ are (i) VDR binding at more than 10,000 sites, (ii) chromatin opening at some 4,500 loci, (iii) changes in CTCF-based TAD anchors affecting some 600 chromatin loops, and (iv) increased PU.1 pioneer factor binding at more than 5,000 regions. This led to the chromatin model of vitamin D signaling (Figure 1). In this model, VDR already binds, in the absence of ligand, to a limited number of loci within accessible chromatin, while 1,25(OH)_2_D_3_ stimulation increases, *via* support of pioneer factors like as PU.1, the number of DNA-bound VDR molecules. This VDR binding leads to changes in chromatin accessibility, which increases the binding strength of TAD anchor forming CTCF sites upstream and downstream of prominent VDR binding loci (41).

Some 300 conserved persistent VDR sites act as key nodes, at which not only primary contacts of VDR ligands with the genome are established, but also functional consequences of vitamin D induction are coordinated throughout the whole stimulation period (13). For more than half of all primary vitamin D target genes a regulatory scenario applies, where each gene is controlled by one or more conserved persistent VDR sites being located within the same TAD. In addition, a few 100 transient VDR sites mediate more tissue-specific primary functions of vitamin D, such as immune system regulation (13). In total, five TAD classes are distinguished that differ in the number of persistent and transient VDR sites and contain sets of genes that represent different physiological functions of vitamin D. Most of the remaining VDR sites are involved in mediating secondary effects of vitamin D.

## *In Vivo* Investigations of Vitamin D Signaling

*In vitro* cell culture models, such as THP-1 cells, use experimental setups that are designed for obtaining maximal effects of 1,25(OH)_2_D_3_ in short time periods, such as 24 h, but may not reflect the reality of the endocrinology of vitamin D *in vivo* (2, 42). In fact, the genetic origin of today’s populations from East Africa and respective minor changes in physiology in the limited time, since the exodus some 50,000 years ago, suggest that humans are still primarily adapted to a constant vitamin D levels rather to changes in 25(OH)D_3_ serum concentration between winter and summer (43). This raises the question of how far results from *in vitro* experiments represent vitamin D’s actions *in vivo*.

The VitDbol vitamin D intervention trial (NCT02063334, ClinicalTrials.gov) studied under *in vivo* conditions vitamin D-dependent gene regulation in humans. From healthy young adults, peripheral blood mononuclear cells (PBMCs) were isolated at days 0, 1, and 2 after supplementation with a vitamin D_3_ bolus. In phase I of VitDbol, changes in chromatin accessibility were measured at selected genomic regions (44) and alterations in gene expression were determined (45). The serum 25(OH)D_3_ concentrations of the subjects raised in average by some 20 nM, i.e., a 20–40% increase in the vitamin D status is sufficient to open chromatin and to activate genes.

VitDbol participants differed significantly both on the level of changes in chromatin accessibility as well as on vitamin D target gene expression (44, 45). Accordingly, they could be segregated into low, mid, and high responders to vitamin D. Together with comparable results from the long-term vitamin D intervention study VitDmet (46), the VitDbol results served as the basis for the concept of the personalized vitamin D index (47). Some of the differences between individuals may be based on variations in their genome, such as SNPs, affecting the vitamin D status (48). However, in analogy to common aging-related disorders, more likely differences in the epigenome of the study participants are the main molecular explanation for alterations in the underlying traits. Accordingly, throughout their entire life a significant proportion of the human population may have a vitamin D status that is significantly lower than the needs of the respective individual for optimal function of vitamin D endocrinology.

In contrast to suggestions from classical pharmacogenetics, an individual’s health or disease status cannot be deduced reliably from a single genotyping experiment (49). Therefore, persons need to be profiled on the level of their epigenome and transcriptome in time series experiments. In phase II of VitDbol, one individual received once a month a vitamin D_3_ bolus three times in a row (50). Figure 2 illustrates changes in chromatin accessibility of PBMCs within 2 days. FAIRE-seq was used to detect accessible chromatin at 5,205 genomic loci, the 853 most prominent of which were categorized into early, delayed, and non-responding genomic regions. Already after 1 day 70 loci showed significant chromatin opening or closing and after 2 days 361 additional genomic sites were affected. Although in PBMCs, the number of chromatin sites with significantly changed accessibility is far lower than THP-1 cells (28), some 85% of the most prominent genomic loci are found both *in vitro* and *in vivo* (50).

The main cellular components of PBMCs, lymphocytes, and monocytes, belong to the adaptive and innate immune system, respectively. This fits well with the observation that the human leukocyte antigen (HLA) region in chromosome 6 is an epigenome “hotspot” in PBMCs (50). Interestingly, the epigenome at the *HLA* cluster is very responsive to vitamin D. This provides a first molecular explanation that how vitamin D may modulate actions of the immune system (51).

## Conclusion

Vitamin D is known as a molecule that controls calcium homeostasis and bone formation, but in humans VDR’s genome-wide actions are investigated primarily in the hematopoietic system. This emphasizes the impact of vitamin D in innate and adaptive immunity. The VitDbol study demonstrated that the human epigenome responds already within 1–2 days to vitamin D. Importantly, the design of VitDbol allows safe human *in vivo* experiments. Nevertheless, such *in vivo* investigations cannot provide the same level of reproducibility than *in vitro* cell culture experiments, in which conditions, such as nutrient availability and temperature, are far more constant.

## Author Contributions

This is a single author mini-review. CC has written all text and created both figures.

## Conflict of Interest Statement

The author declares that the research was conducted in the absence of any commercial or financial relationships that could be construed as a potential conflict of interest.

## Abbreviations

1,25(OH)_2_D_3_, 1α,25-dihydroxyvitamin D_3_; 25(OH)D_3_, 25-hydroxyvitamin D_3_; AGPAT1, 1-acylglycerol-3-phosphate O-acyltransferase 1; ChIP-seq, chromatin immunoprecipitation sequencing; FAIRE-seq, formaldehyde-assisted isolation of regulatory elements sequencing; HLA, human leukocyte antigen; PBMC, peripheral blood mononuclear cell; TAD, topologically associated domain; TSS, transcription start site; VDR, vitamin D receptor.
